# Pan-Cancer Analysis Based on *EPOR* Expression With Potential Value in Prognosis and Tumor Immunity in 33 Tumors

**DOI:** 10.3389/fonc.2022.844794

**Published:** 2022-03-14

**Authors:** Yajing Zhang, Senyu Wang, Songtao Han, Yangchun Feng

**Affiliations:** ^1^ Clinical Laboratory Center, Cancer Hospital Affiliated to Xinjiang Medical University, Xinjiang, China; ^2^ Xinjiang Key Laboratory of Oncology, Cancer Hospital Affiliated to Xinjiang Medical University, Xinjiang, China; ^3^ Clinical Laboratory Center, The Second Hospital Affiliated to Xinjiang Medical University, Xinjiang, China; ^4^ Clinical Laboratory Center, Hospital of Traditional Chinese Medicine Affiliated to Xinjiang Medical University, Xinjiang, China

**Keywords:** erythropoietin receptor, expression, prognosis, tumor immunity, pan-cancer

## Abstract

**Background:**

Erythropoietin receptor (EPOR), a member of the cytokine class I receptor family, mediates erythropoietin (EPO)-induced erythroblast proliferation and differentiation, but its significance goes beyond that. The expression and prognosis of *EPOR* in cancer remain unclear.

**Methods:**

This study intended to perform a pan-cancer analysis of *EPOR* by bioinformatics methods. Several databases such as GTEx, TCGA, CCLE, and others were used to explore the overall situation of *EPOR* expression, and the correlation of *EPOR* expression with prognosis, microRNAs (miRNAs), immune infiltration, tumor microenvironment, immune checkpoint genes, chemokines, tumor mutation burden (TMB), microsatellite instability (MSI), methyltransferases, and DNA mismatch repair (MMR) genes in 33 tumors was analyzed. In addition, we compared the promoter methylation levels of *EPOR* in cancer tissues with those in normal tissues and performed protein–protein interaction network, gene–disease network, and genetic alteration analyses of *EPOR*, and finally enrichment analysis of EPOR-interacting proteins, co-expressed genes, and differentially expressed genes.

**Results:**

The TCGA database showed that *EPOR* expression was upregulated in BLCA, CHOL, HNSC, KIRC, LIHC, STAD, and THCA and downregulated in LUAD and LUSC. After combining the GTEx database, *EPOR* expression was found to be downregulated in 18 cancer tissues and upregulated in 6 cancer tissues. The CCLE database showed that *EPOR* expression was highest in LAML cell lines and lowest in HNSC cell lines. Survival analysis showed that high *EPOR* expression was positively correlated with OS in LUAD and PAAD and negatively correlated with OS in COAD, KIRC, and MESO. Moreover, *EPOR* had a good prognostic ability for COAD, LUAD, MESO, and PAAD and also influenced progression-free survival, disease-specific survival, disease-free survival, and progression-free interval in specific tumors. Further, *EPOR* was found to play a non-negligible role in tumor immunity, and a correlation of *EPOR* with miRNAs, TMB, MSI, and MMR genes and methyltransferases was confirmed to some extent. In addition, the enrichment analysis revealed that *EPOR* is involved in multiple cancer-related pathways.

**Conclusion:**

The general situation of *EPOR* expression in cancer provided a valuable clinical reference. *EPOR* may be target gene of hsa-miR-575, etc. A pan-cancer analysis of panoramic schema revealed that EPOR not only may play an important role in mediating EPO-induced erythroblast proliferation and differentiation but also has potential value in tumor immunity and is expected to be a prognostic marker for specific cancers.

## Introduction

Cancer is a key public health issue worldwide, and the “COVID-19” epidemic that continues from 2019 to the present hinders cancer diagnosis and treatment, which may lead to higher cancer mortality rates (1). Reducing cancer mortality is a lifelong pursuit, so we should continue to strengthen medical research in the field of human cancers. Erythropoietin receptor (EPOR) is a member of the cytokine class I receptor family that mediates erythropoietin (EPO)-induced proliferation and differentiation of erythroblasts, with a structural motif consisting of two extracellular immunoglobulin-like domains, four similarly spaced cysteine residues, and the sequence WSXWS, lacking tyrosine kinase activity and binding to JAK kinase, forming homodimer, heterodimer, or heterotrimer complexes (2). Upon EPO stimulation, binding to its homologous dimer receptor complex induces the activation of Janus kinase 2 (JAK2), a non-receptor tyrosine kinase (3). Activation of JAK2 results in the activation of specific downstream effectors, such as STAT1, STAT3, and STAT5 (4); the signal transducer and activator of transcription 5 (STAT5) usually refers to STAT5A and STAT5B proteins (5) and thus activates phosphatidylinositol 3-kinase (PI3K), protein kinase B (AKT), mitogen-activated protein kinase (MAPK), and extracellular signal-regulated kinase 1/2 (ERK1/2) (6, 7). EPOR was originally discovered and described in erythroid progenitor cells, but it is also present in non-hematopoietic cells (tissues, organs) such as adipose tissue (8), bone progenitor cells (9), neurons (10), endothelial cells (11), and intestinal tract (12). It is also widely present in various cancer cells and tumor tissues, such as head and neck squamous cell carcinoma (HNSC) (13), lymphoid neoplasm diffuse large B-cell lymphoma (DLBC) (14), rhabdomyosarcoma (15), breast cancer (16), liver hepatocellular carcinoma (LIHC) (17), and laryngeal malignancy (18). Studies have shown that it is upregulated in stomach adenocarcinoma (STAD) (19), LIHC (20), prostate adenocarcinoma (PRAD) (21), glioma (22), thyroid carcinoma (THCA) (23), cholangiocarcinoma (CHOL) (24), and other cancers. Seibold et al. (25) concluded that in locally advanced squamous cell carcinoma of the head and neck, EPOR expression was an independent prognostic factor for OS, and improved OS was significantly associated with the absence of EPOR expression. Lin et al. (26) also concluded that in patients with oral squamous carcinoma, high EPOR expression was associated with aggressive tumor behavior and poorer prognosis. Leo et al. (27) concluded that there was no significant difference in overall survival rate and recurrence-free survival rate among patients with different EPOR expression in cervical cancer. Szendrői et al. (28) found that *EPOR* gene expression was associated with a good prognosis in primary renal cell carcinoma. Rózsás et al. (29) concluded that high levels of *EPOR* mRNA in lung adenocarcinoma (LUAD) were associated with significantly increased overall survival rate and that high *EPOR* levels could be used as a potential positive prognostic marker for LUAD. Våtsveen et al. (30) found high levels of *EPOR* mRNA in myeloma cells to be associated with a better survival prognosis and suggested that *EPOR* expression may be a novel prognostic marker in primary myeloma. As such, the impact of *EPOR* expression on the prognosis of cancer patients cannot be determined, and whether it can be a valid prognostic marker for cancer remains to be explored, as well as the lack of studies on *EPOR* in pan-cancer. In recent years, pan-cancer studies have become increasingly popular, and they are more reflective of cancer genes as a whole. In this study, we summarized *EPOR* expression using the GTEx, TCGA, CCLE, HPA, and GEPIA databases. Pan-cancer analysis explored the correlation of *EPOR* expression with prognosis, microRNAs (miRNAs), immune cell infiltration, tumor microenvironment (TME), immune checkpoint genes, chemokines, tumor mutation burden (TMB), microsatellite instability (MSI), methyltransferases, and DNA mismatch repair (MMR) genes in 33 tumors. In addition, we compared the promoter methylation levels of *EPOR* in cancer tissues with normal tissues and performed protein–protein interaction network, gene–disease network, and genetic alteration analyses of *EPOR*, and finally enrichment analyses of EPOR-interacting proteins, co-expressed genes, and differentially expressed genes.

## Materials and Methods

### Differential Expression Analysis of *EPOR*

*EPOR* mRNA expression matrices and clinical information data of each tumor tissue and normal tissue were obtained from the Genotype-Tissue Expression (GTEx) database (https://gtexportal.org/) and The Cancer Genome Atlas (TCGA) database (https://tcga-data.nci.nih.gov/tcga/), and the two databases were integrated. The expression of *EPOR* in 33 tumor tissues (TCGA) was compared with that in adjacent tissues (TCGA) and normal tissues (GTEx) by the Wilcoxon rank-sum test. *EPOR* RNA expression in 55 tissue types, 51 single-cell types, and 69 cell lines was obtained from the Human Protein Atlas (HPA) database (https://www.proteinatlas.org/). The gene expression matrix of each tumor cell line was downloaded from the Cancer Cell Line Encyclopedia (CCLE) database (https://portals.broadinstitute.org/ccle), and expression analysis was performed. Box plots/bar charts were plotted by the R package ggplot2. To evaluate the differences in *EPOR* expression in different tumor stages, we used the “Expression DIY” module of the Gene Expression Profiling Interactive Analysis (GEPIA) database (http://gepia.cancer-pku.cn/index.html) to plot the pathological stages (I, II, III, and IV) of *EPOR* in TCGA tumor types.

### Prognostic Analysis of *EPOR* in Pan-Cancer

The association between *EPOR* expression and overall survival (OS) based on all tumor samples was explored in 33 cancer datasets selected from the “Survival” module of GEPIA database. In order to further explore the prognosis of *EPOR* expression in patients with each type of cancer, we obtained clinical information on pan-cancer from the TCGA database and the expression levels of *EPOR* in tumor and adjacent tissues were divided into high expression group and low expression group by the dichotomy method. The correlation between *EPOR* expression and OS, progression-free survival (PFS), disease-specific survival (DSS), disease-free survival (DFS), and progression-free interval (PFI) in pan-cancer was analyzed by univariate Cox regression and visualized by R package forest plot. The Kaplan–Meier (KM) method and log-rank test were used for survival analysis, and then survival curves were plotted using the R packages survminer and survival. To further explore the predictive ability of *EPOR*, receiver operating characteristic (ROC) curve analysis was performed with the false positive rate (FPR) as the horizontal coordinate and the true positive rate (TPR) as the vertical coordinate. The closer the AUC is to 1, the better the predictive ability, with some accuracy at AUC of 0.7 to 0.9 and high accuracy at AUC above 0.9. A ROC analysis was performed by R package pROC, time-dependent ROC analysis was performed by timeROC, and R package ggplot2 was visualized.

### Protein–Protein Interaction, Gene–Disease Network, *EPOR*–miRNA Correlation, and Genetic Alteration Analyses

We used the STRING database (https://string-db.org/) and GPS-Prot online server (http://gpsprot.org/index.php) respectively including protein–protein interaction (PPI) network analysis on EPOR and obtained the intersection of interacting proteins. A gene–disease network analysis of *EPOR* based on genetic association was performed using the OPENTARGET platform with a minimum score set at 0.4. A correlation analysis between *EPOR* expression and miRNAs by miRDB (http://www.mirdb.org/), TargetScanHuman (http://www.targetscan.org/vert_80/), and miRWalk (http://129.206.7.150/) databases was performed. The score was set as 1, and the binding site of miRNA and *EPOR* mRNA was set as 3UTR in the miRWalk database. The closer the context++ score percentile is to 100, the greater the probability that the site is a true target in the TargetScanHuman database. In addition, we used cBioPortal for Cancer Genomics (https://www.cbioportal.org/) to explore the genetic alterations of *EPOR* in pan-cancer. 33 cancer datasets (TCGA, PanCancer Atlas) were selected to analyze the types and frequencies of alterations in *EPOR* genes in pan-cancer by the “OncoPrint” module and “Cancer Types Summary” module. OS, PFS, and DFS of *EPOR* genes in altered and unaltered groups were analyzed by the “Comparison/Survival” module.

### Correlation Analysis of *EPOR* With Immune Cells and Tumor Microenvironment

We used the tumor immune assessment resource 2.0 (TIMER2.0) server (http://timer.cistrome.org/) to analyze the correlation of *EPOR* expression with six types of immune cells, B cells, CD4 + T cells, CD8 + T cells, macrophages, neutrophils, and dendritic cells (DCs), and tumor purity in 32 cancers (except LAML). The immune cells here are derived from tumor-infiltrating immune cells. To further explore the correlation of *EPOR* with immune cells, tumor RNA-seq data and mRNA expression data of paired normal tissue samples were downloaded from TCGA database. In addition to the TIMER algorithm, the more comprehensive assessment of immune cell infiltration was performed by the R package immunedeconv’s CIBERSORT, EPIC, quanTIseq, xCell, and MCP-counter algorithms. The tumor microenvironment (TME) can be evaluated by immune score, stromal score, and estimate score, which indicate tumor immune cell infiltration, presence of tumor tissue mesenchyme, and tumor purity, respectively. The correlation of *EPOR* expression with TME was analyzed using the R package estimate.

### Correlation Analysis of *EPOR* With Immune Checkpoints and Chemokines

To investigate the correlation between *EPOR* gene expression and immune checkpoints in pan-cancer, we extracted the expression profile data of more than 30 common immune checkpoints from TCGA database and calculated and analyzed the correlation between them using Spearman’s rank correlation coefficient. In addition, we explored the correlation of *EPOR* expression with 41 chemokines through TISIDB (http://cis.hku.hk/TISIDB/), a portal for tumor–immune system interactions.

### Correlation Analysis of *EPOR* With Tumor Mutation Burden and Microsatellite Instability

Tumor mutation burden (TMB) is defined as the total number of substitutions and insertions/deletions per megabase in the exon-coding regions of the genes evaluated in a tumor sample, in short, the total number of mutations present in the tumor. Microsatellite instability (MSI) is a phenomenon in which the length of a microsatellite is altered and a new microsatellite allele appears in a tumor due to insertion or deletion of a repeat unit compared to normal tissue. MAF files for all cancers were downloaded from TCGA database and calibrated by dividing by the exon region size to calculate the TMB. MSI scores were also available from TCGA database. The correlation of *EPOR* expression with TMB or MSI was then analyzed using Spearman analysis.

### Methylation Level of *EPOR* and Correlation With Methyltransferases and DNA Mismatch Repair Genes

Methylation is the process of catalytic transfer of methyl groups from active methyl compounds to other compounds. We analyzed the promoter methylation levels of *EPOR* in tumors and paired normal tissues by the “TCGA” module of the UALCAN cancer OMICS database (http://ualcan.path.uab.edu/), expressed by beta value ranging from 0 (unmethylated) to 1 (fully methylated). A different beta value cutoff has been considered to indicate hypermethylation [beta value: 0.7–0.5] or hypomethylation [beta value: 0.3–0.25]. Meanwhile, we used TCGA expression profile data to analyze the correlation between *EPOR* expression and the expression of 33 methyltransferases, including N6-methyladenosine (m6A), 5-methylcytidine (m5C), N1-methyladenosine (m1A), 7-methylguanine (m7G), and 2′-0-methylation modification-related methyltransferases. DNA mismatch repair (MMR) genes are a class of genes related to the mismatch repair response in humans, which mainly repair base mismatches during DNA replication and play an important role in maintaining the stability of the genome. We analyzed the correlation of *EPOR* expression with five MMR genes, *MLH1* (*MutL homolog 1*), *MSH2* (*MutS homologue 2*), *MSH6* (*MutS homologue 6*), *PMS2* (*PMS1 homologue 2*), and *EPCAM* (*Epithelial cell adhesion molecular*), using TCGA expression profile data.

### Enrichment Analysis of Interacting Proteins, Co-Expressed Genes, and Differentially Expressed Genes

We obtained the strongest positively correlated co-expressed genes with *EPOR* from the Oncomine database (https://www.oncomine.org/resource/login.html), with a p-value set to 1E-4, fold change set to 2, and gene rank set to TOP 10%. A total of 210 genes were obtained by merging the corresponding genes of the previously obtained interacting proteins. These 210 genes were enriched by Gene Ontology (GO) term and Kyoto Encyclopedia of Genes and Genomes (KEGG) pathway to explore the potential biological functions of EPOR-interacting proteins and co-expressed genes in pan-cancer, and GO enrichment analysis included molecular function (MF), cellular component (CC), and biological process (BP). RNA-seq data for *EPOR* were obtained from TCGA database, and differentially expressed gene (DEG) analysis was performed by the R package DESeq2. Gene Set Enrichment Analysis (GSEA) was used to explore the potential biological functions of DEGs in pan-cancer, and a normalized enrichment score (NES) > 1.5, false discovery rate (FDR) < 0.25, and p.adjust < 0.05 were considered significantly enriched; an upward-of-the-curve peak indicated positive regulation, and a downward-of-the-curve peak indicated negative regulation. GO, KEGG analysis, and GSEA were all performed by the R package ClusterProfiler.

### Statistical Analysis

Box plots/bar charts were plotted by the R package ggplot2. In survival analysis, the KM method and log-rank test were used to analyze patient prognosis based on univariate Cox regression analysis and different expression levels of *EPOR*; the R package forest plot was used to plot forest plots, and R packages survminer and survival were used to plot survival curves. ROC analysis was performed by R package pROC, and time-dependent ROC analysis was performed by timeROC and visualized by R package ggplot2. R package immunedeconv was used to assess immune cell infiltration. The correlation of *EPOR* expression with TME was analyzed by R package estimate. The correlation of *EPOR* expression with TME, immune checkpoints, chemokines, TMB, MSI, methyltransferases, and DNA mismatch repair genes was evaluated by Spearman correlation analysis. Correlation heat maps were visualized with the R package ggplot. Radar plots were performed using the ggradar package and the ggplot2 package. Each p-value < 0.05 was considered statistically significant. Correlations were considered significant when p < 0.05 and |R| > 0.20.

## Results

### *EPOR* Expression Profile

**Figure 1:**
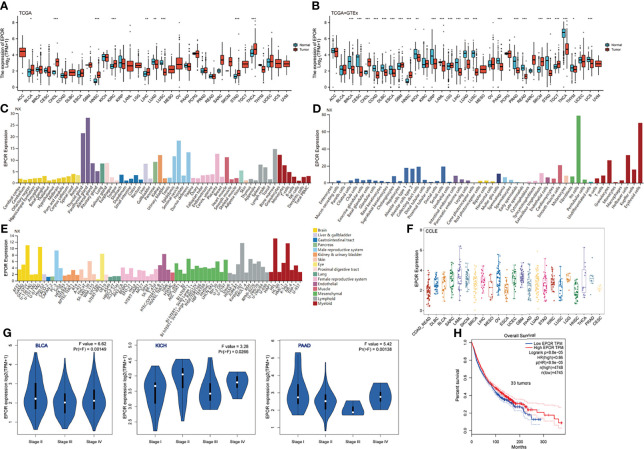
*EPOR* expression profile. **(A)** Differential *EPOR* expression levels in 33 tumors and adjacent normal tissues in TCGA database, *p < 0.05, **p < 0.01, ***p < 0.001. **(B)** Differential *EPOR* expression levels in 33 tumors and normal tissues in TCGA database and GTEx database, *p < 0.05, **p < 0.01, ***p < 0.001. **(C)** *EPOR* expression levels in various organs. Consensus normalized expression (NX) levels for 55 tissue types and 6 blood cell types, created by combining the data from the three transcriptomics datasets (HPA, GTEx, and FANTOM5) using the internal normalization pipeline. **(D, E)** *EPOR* normalized expression (NX) levels for 51 single-cell types and 69 cell lines in the HPA database. **(F)** *EPOR* expression levels in cell lines of 23 tumors in the CCLE database. **(G)** Violin plots showing differential *EPOR* expression levels (log2 TPM + 1) between pathological stages (stages I, II, III, and IV). Only TCGA cancers with statistically significant differences between the pathological stages are presented. **(H)** Prognosis of *EPOR* expression for OS from 33 cancer datasets in the GEPIA database.

Analysis of TCGA dataset showed that the expression levels of *EPOR* in bladder urothelial carcinoma (BLCA), CHOL, HNSC, kidney renal clear cell carcinoma (KIRC), LIHC, STAD, and THCA were higher than normal tissues but those in LUAD and lung squamous cell carcinoma (LUSC) were lower than normal tissues (**Figure 1A**), as detailed in **Supplementary Table 1**. Because TCGA database has lesser normal tissue data (n = 727), we combined the normal tissue data of GTEx database (n = 7,568), compared with the tumor data of TCGA database (n = 9,807), and the results showed that the expression levels of *EPOR* were lower than normal tissues in 18 cancer tissues including breast invasive carcinoma (BRCA), cervical squamous cell carcinoma and endocervical adenocarcinoma (CESC), and colon adenocarcinoma (COAD), and higher than normal tissues in CHOL, glioblastoma multiforme (GBM), HNSC, brain lower grade glioma (LGG), skin cutaneous melanoma (SKCM), and testicular germ cell tumor (TGCT) (**Figure 1B**); the details are shown in **Supplementary Table 2**. In addition, we explored the expression of *EPOR* in 55 tissue types, 51 single-cell types, and 69 cell lines in the HPA database, which showed enhanced specificity in erythroid cells, hepatic stellate cells, and Hofbauer cells, and both tissue and cell line specificity were low (**Figures 1C–E** **)**. The CCLE database (n = 765) showed the expression of *EPOR* in 23 tumor cell lines, which was the highest in acute myeloid leukemia (LAML) cell lines, the lowest in HNSC cell lines, the highest in the MOLM.16 cell line of LAML, and the lowest in the HCC1187 cell line of BRCA (**Figure 1F** and **Supplementary Table 3**). The GEPIA database showed that *EPOR* expression was significantly different only among different tumor stages in BLCA, kidney chromophobe (KICH), and pancreatic adenocarcinoma (PAAD), and *EPOR* expression was not associated with tumor stage in the other 30 cancers (**Figure 1G**).

### Prognosis of *EPOR* Expression in Pan-Cancer

**Figure 2:**
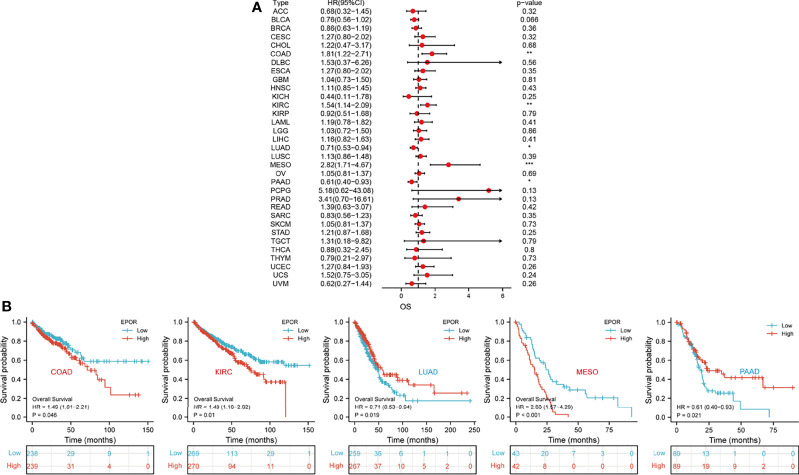
The correlation between *EPOR* expression and prognosis for OS in pan-cancer. **(A)** The correlation between *EPOR* expression and OS in different cancer types of TCGA. The red part represents the risk ratio. *p < 0.05, **p < 0.01, ***p < 0.001. **(B)** Kaplan–Meier analysis was used to generate the survival curve of tumors with significant correlation between *EPOR* expression and OS.

**Figure 3:**
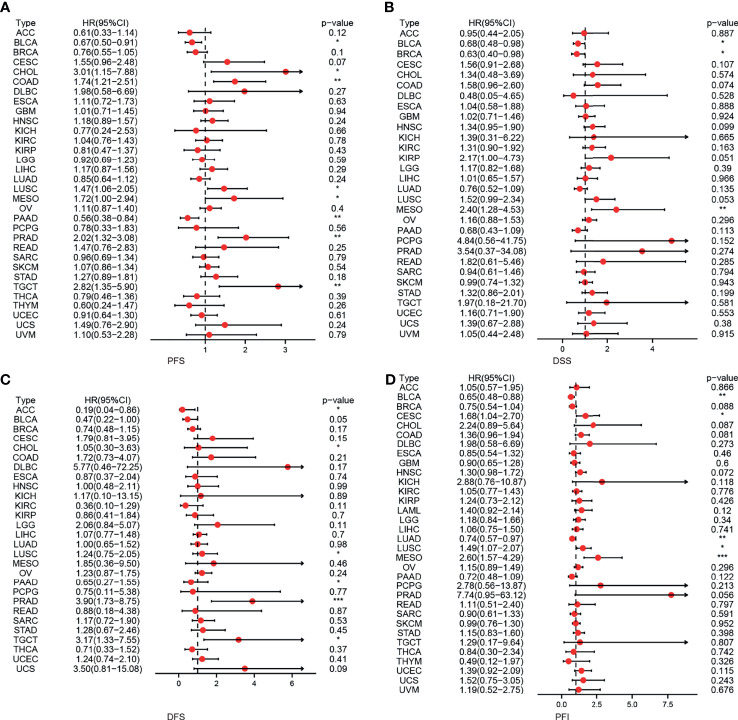
The correlation between *EPOR* expression and prognosis for PFS, DSS, DFS, and PFI in pan-cancer. **(A)** The correlation between *EPOR* expression and PFS in different cancer types of TCGA. **(B)** The correlation between *EPOR* expression and DSS in different cancer types of TCGA. **(C)** The correlation between *EPOR* expression and DFS in different cancer types of TCGA. **(D)** The correlation between *EPOR* expression and PFI in different cancer types of TCGA. The red part represents the risk ratio. *p < 0.05, **p < 0.01, ***p < 0.001.

**Figure 4:**
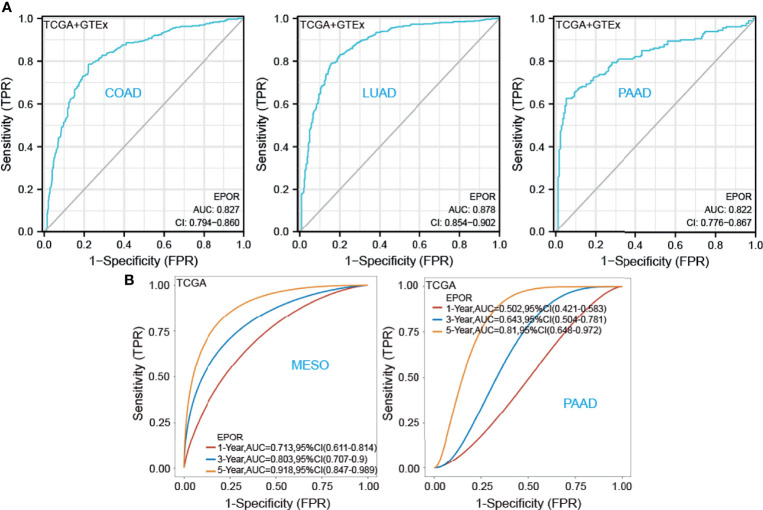
To further explore the predictive ability of *EPOR* on the prognosis of patients with COAD, KIRC, LUAD, MESO, and PAAD. **(A)** ROC analysis showed that *EPOR* had better predictive ability on the prognosis of COAD, LUAD, and PAAD in TCGA and GTEx databases. **(B)** Time-dependent ROC analysis showed that *EPOR* had better predictive ability for 1-, 3-, and 5-year survival rate in MESO patients and for 5-year survival in PAAD patients.

First, we used 33 cancer datasets as the study unit (n = 9,493) and the *EPOR* high-expression group had a longer OS compared to the *EPOR* low-expression group (**Figure 1H**). We further explored the prognostic impact of *EPOR* on patients with each type of cancer, using gene expression profile data and univariate regression analysis to plot forest plots, and KM survival curves for tumors with significantly affected OS. OS showed that *EPOR* was significantly associated with the prognosis of COAD, KIRC, LUAD, mesothelioma (MESO), and PAAD (**Figure 2A**), where a high *EPOR* expression was associated with a low survival rate in COAD (p = 0.046), KIRC (p = 0.01), and MESO (p < 0.001); a high *EPOR* expression was associated with high survival rate in LUAD (p = 0.019) and PAAD (p = 0.021), suggesting that a high *EPOR* expression may be a risk factor for poor prognosis in patients with COAD, KIRC, and MESO, and a low *EPOR* expression may be a risk factor for poor prognosis in patients with LUAD and PAAD (**Figure 2B**). The PFS showed that *EPOR* was significantly associated with the prognosis of BLCA (HR = 0.67, p < 0.05), PAAD (HR = 0.56, p < 0.01), CHOL (HR = 3.01, p < 0.05), COAD (HR = 1.74, p < 0.01), LUSC (HR = 1.47, p < 0.05), MESO (HR = 1.72, p < 0.05), PRAD (HR = 2.02, p < 0.01), and TGCT (HR = 2.82, p < 0.01) (**Figure 3A**). The DSS showed that *EPOR* was significantly associated with the prognosis of BLCA (HR = 0.68, p < 0.05), BRCA (HR = 0.63, p < 0.05), and MESO (HR = 2.40, p < 0.01) (**Figure 3B**). The DFS showed that *EPOR* was significantly associated with the prognosis of adrenocortical carcinoma (ACC) (HR = 0.19, p < 0.05), PAAD (HR = 0.65, p < 0.05), CHOL (HR = 1.05, p < 0.05), LUSC (HR = 1.24, p < 0.05), PRAD (HR = 3.90, p < 0.01), and TGCT (HR = 3.17, p < 0.05) (**Figure 3C**). The PFI showed that *EPOR* was significantly associated with the prognosis of BLCA (HR = 0.65, p < 0.01), LUAD (HR = 0.74, p < 0.01), CESC (HR = 1.68, p < 0.05), LUSC (HR = 1.49, p < 0.05), and MESO (HR = 2.60, p < 0.001) (**Figure 3D**). Among them, OS and PFI consistently showed that *EPOR* was a protective factor for LUAD prognosis; OS, PFS, and DFS consistently showed that *EPOR* was a protective factor for PAAD prognosis; OS and PFS consistently showed that *EPOR* was a risk factor for COAD prognosis; OS, PFS, DSS, and PFI consistently showed that *EPOR* was a risk factor for MESO prognosis; PFS, DSS, and PFI consistently showed that *EPOR* was a protective factor for BLCA prognosis; PFS and DFS consistently showed that *EPOR* was a risk factor for CHOL, PRAD, and TGCT prognosis; and PFS, DFS, and PFI consistently showed that *EPOR* was a risk factor for LUSC prognosis. In addition, to further explore the predictive ability of *EPOR* on the prognosis of patients with COAD, KIRC, LUAD, MESO, and PAAD, we first performed ROC analysis jointly with TCGA and GTEx databases and unexpectedly found that *EPOR* had better predictive ability on the prognosis of COAD, LUAD, and PAAD, with AUCs above 0.8 (**Figure 4A**). Subsequently, we only analyzed the data from TCGA database, and the time-dependent ROC analysis showed that *EPOR* had better predictive ability for 1-, 3-, and 5-year survival rate in MESO patients (AUC = 0.713, 0.803, 0.918) and for 5-year survival in PAAD patients (AUC = 0.81) (**Figure 4B**). The parts not shown in **Figure 4** are shown in **Supplementary Figure 1**.

### Interacting Protein Network, Gene–Disease Network, and *EPOR*–miRNA Correlation Analyses

**Figure 5:**
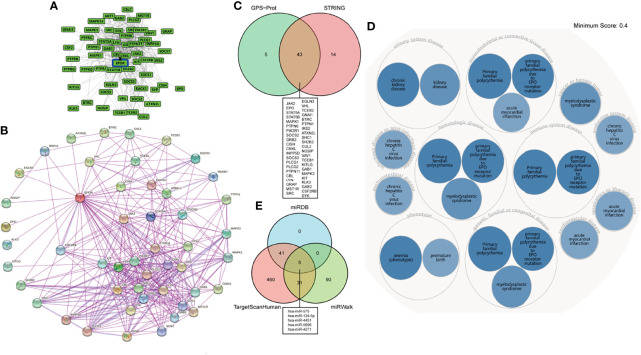
**(A)** EPOR-interacting proteins network analysis in the GPS-Prot online server. **(B)** EPOR-interacting proteins network analysis in the STRING database. **(C)** Venn diagram showed the intersection of EPOR-interacting proteins from GPS-Prot and STRING. **(D)** The OPENTARGET platform was used to conduct a gene–disease network analysis of *EPOR*. **(E)** Venn diagram showed miRNAs associated with *EPOR* genes from miRDB, TargetScanHuman, and miRWalk databases.

To explore the EPOR-interacting proteins in pan-cancer, we used the GPS-Prot online server (**Figure 5A**) and the STRING database (**Figure 5B**), respectively. GPS-Prot unites many databases such as DIP, BioGrid, HPRD, IntACT, MINT, BIND, and MIPS. The results showed that EPOR interacted with various proteins such as JAK2, EPO, STAT5A/B, MAPK1/3, and SOCS2/3 (**Figure 5C**). To explore the diseases associated with the *EPOR* gene, we used the OPENTARGET platform, and the results showed that *EPOR* is associated with numerous diseases such as urological diseases, musculoskeletal or connective tissue diseases, immune system diseases, endocrine system diseases, hematological system diseases, and cancer or benign tumors (**Figure 5D**). In order to analyze the correlation between *EPOR* expression and miRNAs and find miRNAs that may be involved in posttranscriptional regulation of *EPOR* mRNA, we used the miRDB, TargetScanHuman, and miRWalk databases. The results showed that *EPOR* was the target gene of hsa-miR-575, hsa-miR-134-5p, hsa-miR-4451, hsa-miR-5696, and hsa-miR-4271 in the three databases (**Figure 5E**). In miRDB, when the target score was 100, the corresponding miRNA was hsa-miR-5011-5p. In TargetScanHuman, the context++ score percentile of 10 miRNAs including hsa-miR-939-3p was 99, but the sites were poorly conserved; when the sites were conserved, hsa-miR-503-5p and hsa-miR-335-5p had the higher context++ score percentile (88). The details of the three databases are shown in **Supplementary Figure 2** and **Supplementary Tables 4, 5**.

### Correlation of *EPOR* Expression With Immune Infiltration and Tumor Microenvironment in Pan-Cancer

**Figure 6:**
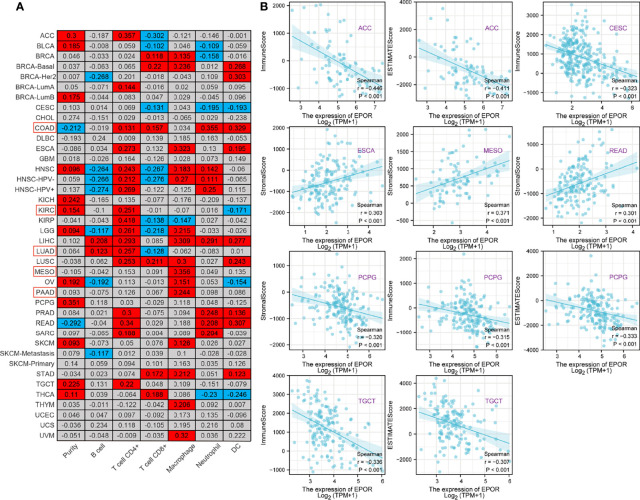
Correlation of *EPOR* expression with immune infiltration and tumor microenvironment in pan-cancer. **(A)** Correlation of *EPOR* expression with six infiltrating immune cells (B cells, CD4 + T cells, CD8 + T cells, macrophages, neutrophils, and dendritic cells) and tumor purity in the TIMER2.0 database. Positive correlation (p < 0.05) in the red part and negative correlation (p < 0.05) in the blue part. **(B)** Correlation of *EPOR* expression with immune score, stromal score, and estimate score in pan-cancer. The part of “|r| ≥ 0.3, p < 0.001” was presented.

To explore the correlation between *EPOR* gene expression and immune infiltration, we first analyzed the correlation of *EPOR* with six infiltrating immune cells (B cells, CD4 + T cells, CD8 + T cells, macrophages, neutrophils, and dendritic cells) and tumor purity in the TIMER2.0 database. The results are shown in **Figure 6A**, with a positive correlation (p < 0.05) in the red part and a negative correlation (p < 0.05) in the blue part. Among them, *EPOR* expression was negatively correlated with tumor purity in COAD (r = -0.212) and positively correlated with tumor purity in KIRC (r = 0.154); *EPOR* expression was negatively correlated with CD8 + T cell expression in LUAD (r = -0.128) and negatively correlated with dendritic cell expression in KIRC (r = -0.171). *EPOR* expression was positively correlated with infiltration of CD4 + T cells (r = 0.131), CD8 + T cells (r = 0.157), neutrophils (r = 0.355), and dendritic cells (r = 0.329) in COAD. It was positively correlated with infiltration of B cells (r = 0.123) and CD4 + T cells (r = 0.257) in LUAD, with CD4 + T cell infiltration in KIRC (r = 0.251), and positively correlated with macrophage infiltration in MESO and PAAD (r = 0.356, 0.244). In addition, the correlation coefficient r between *EPOR* expression and tumor purity exceeded 0.3 in pheochromocytoma and paraganglioma (PCPG). Moreover, *EPOR* expression correlated more strongly with CD4 + T cell infiltration in ACC, kidney renal papillary cell carcinoma (KIRP), and rectum adenocarcinoma (READ) (r = 0.357, 0.418, 0.34); *EPOR* expression correlated more strongly with dendritic cell infiltration in ESCA, LIHC, and uveal melanoma (UVM) (r = 0.323, 0.309, 0.32); and *EPOR* expression correlated more strongly with macrophage infiltration in READ (r = 0.307). To explore the correlation between the levels of infiltration of more types of immune cell subtypes and *EPOR* expression, we conducted a more in-depth study using the CIBERSORT, EPIC, quanTIseq, xCell, and MCP-counter algorithms, which confirmed a degree of correlation between *EPOR* expression and immune cell infiltration in pan-cancer (**Supplementary Figures 3, 4**). The tumor microenvironment influences the occurrence and progression of tumors; in order to further assess the role of *EPOR* in the tumor microenvironment, we explored the correlation of *EPOR* with three scores. The results showed that *EPOR* expression was positively correlated with StromalScore and ImmuneScore for 6 cancers, negatively correlated with StromalScore for 14 cancers, positively correlated with ESTIMATEScore for 5 cancers, and negatively correlated with ImmuneScore and ESTIMATEScore for 16 cancers (**Supplementary Table 6**). Among them, *EPOR* expression in ACC (r = - 0.446), CESC (r = - 0.323), PCPG (r = - 0.315), and TGCT (r = - 0.336) correlated more strongly with ImmuneScore; *EPOR* expression in ACC (r = - 0.411), PCPG (r = - 0.333), and TGCT (r = - 0.307) correlated more strongly with ESTIMATEScore; and *EPOR* expression in ESCA (r = 0.303), MESO (r = 0.371), READ (r = 0.301), and PCPG (r = - 0.320) correlated more strongly with StromalScore (P < 0.001) (**Figure 6B**).

### Correlation of *EPOR* Expression With Immune Checkpoints and Chemokines in Pan-Cancer

**Figure 7:**
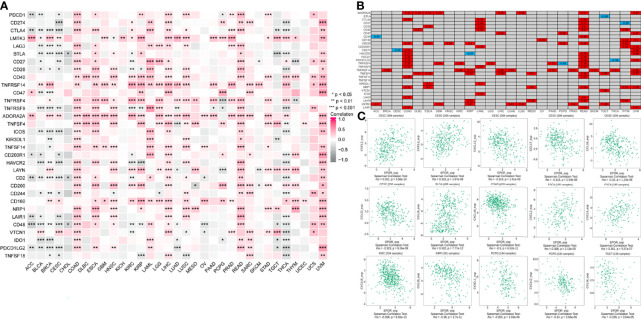
Correlation of *EPOR* expression with immune checkpoints and chemokines in pan-cancer. **(A)** Correlation of *EPOR* expression and more than 30 common immune checkpoint genes in pan-cancer. *p < 0.05, **p < 0.01, ***p < 0.001. **(B)** The stronger correlation between *EPOR* expression and immune checkpoint genes was shown (|r| ≥ 0.3, p < 0.001), with a positive correlation in red and a negative correlation in blue. **(C)** Correlation of *EPOR* expression with 41 chemokines in TISIDB database. The part of “|r| ≥ 0.3, p < 0.001” was presented.

The correlation analysis of *EPOR* expression with immune checkpoint gene expression showed (**Figure 7A**) that *EPOR* expression was positively correlated with 27 immune checkpoint genes in COAD, positively correlated with 10 immune checkpoint genes and negatively correlated with 9 immune checkpoint genes in KIRC, positively correlated with 4 immune checkpoint genes and negatively correlated with 6 immune checkpoint genes in LUAD, and positively correlated with 4 immune checkpoint genes and negatively correlated with 1 immune checkpoint gene in MESO and PAAD. In addition, more than 2/3 of the immune checkpoint genes were associated with *EPOR* expression in ESCA, LIHC, READ, THCA, and UVM. Among 33 tumors, *TNFRSF14* was significantly associated with *EPOR* expression in 20 cases, and *ADORA2A* was significantly associated with *EPOR* expression in 22 cases. The stronger correlation is shown in **Figure 7B** (|r| ≥ 0.3, p < 0.001), with a positive correlation in red and a negative correlation in blue. The correlation analysis of *EPOR* expression with chemokines (**Figure 7C**) showed that *EPOR* expression correlated with CXCL2–3, CXCL13, and CCL17/19/22 in CESC, CCL15 in BLCA, CXCL14 in COAD, CXCL2/5 in ESCA, CXCL16 in KIRC, CCL15 in KIRP,and CX3CL1 and CXCL12 in PCPG, and CCL24 in TGCT showed a strong correlation in expression (|r| ≥ 0.3, p < 0.001).

### Genetic Alterations of *EPOR* in Pan-Cancer, and Correlation of *EPOR* With Tumor Mutation Burden and Microsatellite Instability

**Figure 8:**
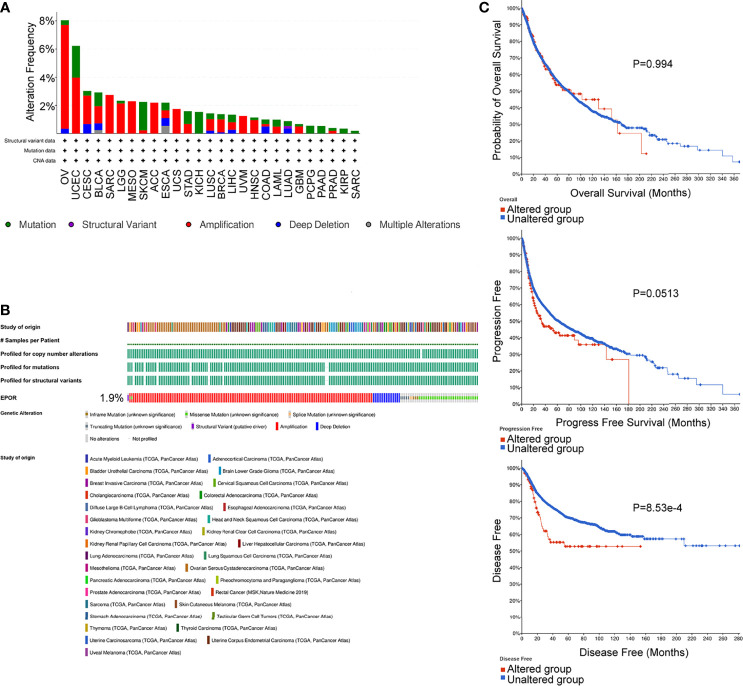
Genetic alterations of *EPOR* in pan-cancer, analyzed by the cBioPortal database. **(A, B)** The types and frequencies of alterations in *EPOR* genes were analyzed by the “OncoPrint” module and “Cancer Types Summary” module. **(C)** OS, PFS, and DFS of *EPOR* genes in altered and unaltered groups were analyzed by the “Comparison/Survival” module.

**Figure 9:**
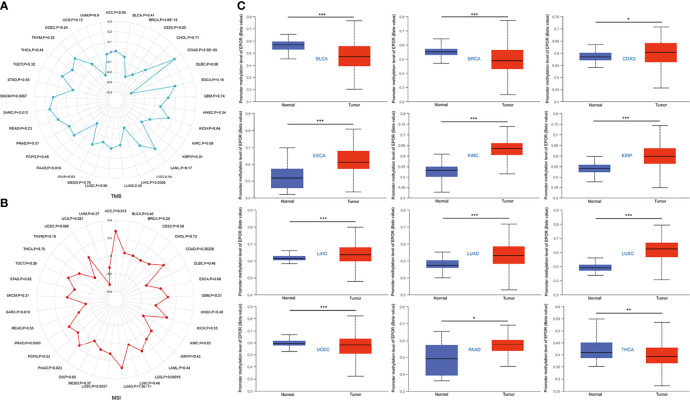
**(A)** Correlation between *EPOR* expression in pan-cancer and TMB described using Spearman’s rank correlation coefficient. **(B)** Correlation between *EPOR* expression in pan-cancer and MSI described using Spearman’ s rank correlation coefficient. **(C)** Boxplots showed differential *EPOR* promoter methylation levels (beta values) between tumor and adjacent normal tissues across TCGA database. *p < 0.05, **p < 0.01, ***p < 0.001.

The cBioPortal database showed that *EPOR* was genetically altered in 27 of 33 cancers, and gene amplification was the most common type of genetic alteration in *EPOR*, with the highest frequency of *EPOR* alteration in OV at around 8% (**Figure 8A**), with an average alteration frequency of 1.9% (**Figure 8B**). Genetic alterations prolonged DFS in patients (p = 8.53e-4) and had no effect on OS and PFS (**Figure 8C**). TMB is an important biomarker for the total number of mutations within a tumor. MSI is also more common in tumors, and it may also serve as a new important biomarker. The analysis showed that *EPOR* expression was positively correlated with the TMB of COAD (p = 2.5e - 05), sarcoma (SARC) (p = 0.012), and SKCM (p = 0.0067) and negatively correlated with the TMB of BRCA (p = 3.6e - 13), CESC (p = 0.03), KIRP (p = 0.01), LIHC (p = 0.0006), and PAAD (p = 0.019) (**Figure 9A**). *EPOR* expression was positively correlated with the MSI of ACC (p = 0.013), COAD (p = 0.00026), LGG (p = 0.00019), LUAD (p = 7.3e - 11), LUSC (p = 0.0057), PAAD (p = 0.023), and PRAD (p = 0.0093) and negatively correlated with the MSI of KIRC (p = 0.02) and SARC (p = 0.019) (**Figure 9B**).

### Methylation Levels of *EPOR* in Pan-Cancerous and Normal Tissues, and Correlation of *EPOR* Expression With Methyltransferases and DNA Mismatch Repair Genes in Pan-Cancerous Tissues

**Figure 10:**
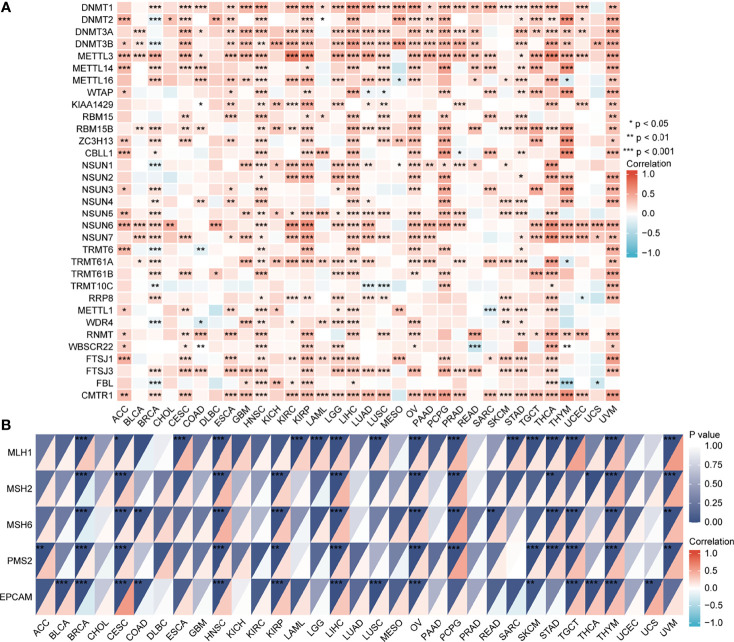
The correlation of *EPOR* gene expression with 33 methyltransferases and 5 DNA mismatch repair genes. **(A)** The correlation of *EPOR* gene expression with 33 methyltransferases, including N6-methyladenosine (m6A), 5-methylcytidine (m5C), N1-methyladenosine (m1A), 7-methylguanine (m7G), and 2′-0-methylation modification-related methyltransferases. **(B)** The correlation of *EPOR* gene expression with 5 DNA mismatch repair genes, including MLH1, MSH2, MSH6, PMS2, and EPCAM genes. *p < 0.05, **p < 0.01, ***p < 0.001.

The UALCAN database showed that promoter methylation levels of *EPOR* were higher in COAD, ESCA, KIRC, KIRP, LIHC, LUAD, LUSC, and PAAD cancer tissues than in normal tissues, and lower in BLCA, BRCA, uterine corpus endometrial carcinoma (UCEC), and THCA cancer tissues than in normal tissues (**Figure 9C**). Subsequently, we explored the relationship between 33 methyltransferases associated with m6A, m5C, m1A, m7G, and 2′-0-methylation modifications and *EPOR*, which contained DNA methyltransferases and RNA methyltransferases. The results showed that *EPOR* was significantly associated with three DNA methyltransferases, DNMT1 (DNA methyl transferase 1), DNMT3A, and DNMT3B, and two RNA methyltransferases, METTL3 (methyltransferase like 3) and CMTR1 (cap methyltransferase 1), in more than 2/3 of the tumors (**Figure 10A**). Among COAD, KIRC, LUAD, MESO, and PAAD, the parts with a stronger correlation between *EPOR* and methyltransferases are shown in **Supplementary Figure 5** (r ≥ 0.3, p < 0.001). MMR genes are able to maintain genomic stability and thus have an impact on cancer development and progression; therefore, we investigated the correlation of five MMR genes with *EPOR*. The results showed that *EPOR* expression was significantly correlated with *MLH1*, *MSH2*, *MSH6*, *PMS2*, and *EPCAM* in BRCA, CESC, HNSC, LIHC, ovarian serous cystadenocarcinoma (OV), and thymoma (THYM), and with four of the MMR genes in KIRP, PCPG, STAD, TGCT, and UVM (**Figure 10B**), and the parts with stronger correlation in pan-cancer are shown in **Supplementary Figure 6** (r ≥ 0.3, p < 0.001).

### Enrichment Analysis of Interacting Proteins, Co-Expressed Genes, and Differentially Expressed Genes

**Figure 11:**
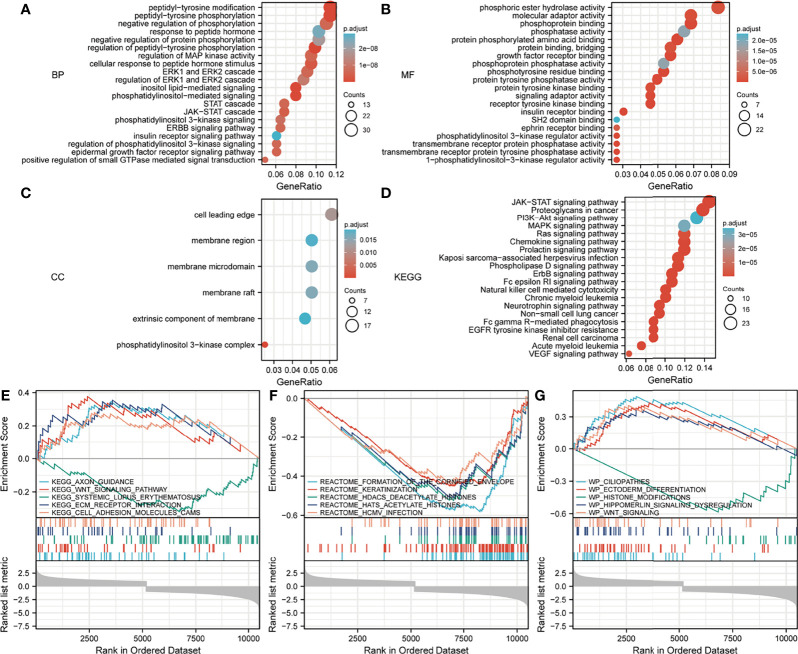
Analysis of the *EPOR* gene enrichment in pan-cancer. **(A)** Top 20 terms in BPs from GO enrichment analysis. **(B)** Top 20 terms in MFs from GO enrichment analysis. **(C)** Top 20 terms in CCs from GO enrichment analysis. **(D)** KEGG enrichment analysis showed the top 20 KEGG pathways. **(E)** Top 5 KEGG pathways from GSEA. **(F)** Top 5 reactome pathways from GSEA. **(G)** Top 5 wiki pathways from GSEA.

Regarding the GO and KEGG enrichment analyses of EPOR-interacting proteins and co-expressed genes, we showed the top 20 enrichment results in **Figure 11**. In BPs, the response to peptide hormone, regulation of MAP kinase activity (MAPK), ERK1 and ERK2 cascade, JAK-STAT cascade, and regulation of phosphatidylinositol 3-kinase (PI3K) signaling dominated (**Figure 11A**). MFs were significantly enriched in phosphoric ester hydrolase activity, protein binding, bridging, protein tyrosine phosphatase activity, receptor tyrosine kinase binding, and PI3K regulator activity (**Figure 11B**). In CCs, they were mainly located in the cell leading edge, membrane region, membrane microdomain, membrane raft, extrinsic component of membrane, and the PI3K complex (**Figure 11C**). KEGG enrichment analysis revealed that EPOR-interacting proteins and co-expressed genes were significantly enriched in JAK-STAT, PI3K-Akt, MAPK, Ras, chemokines, neurotrophic factor signaling pathways, natural killer cell cytotoxic activity, and cancer pathways (**Figure 11D**). GSEA of DEGs showed significant enrichment in KEGG pathways such as axon guidance, Wnt signaling pathway, systemic lupus erythematosus, ECM receptor interactions, and cell adhesion molecules (CAMs) (**Figure 11E** and **Table 1**), with significant enrichment in the formation of the cornified envelope, keratinization, HDAC deacetylate histones, hat acetylate histones, HCMV infection, and other reactome pathways (**Figure 11F** and **Table 2**), with significant enrichment in ciliopathies, ectoderm differentiation, histone modification, Hippo-Merlin signaling dysregulation, Wnt signaling, and other wiki pathways (**Figure 11G** and **Table 3**).

**Table 1:** The information of KEGG pathways from GSEA enrichment analysis.

Term	ES	NES	NOM p-val	FDR q-val	FWER p-val
KEGG_AXON_GUIDANCE	0.3466	2.3891	0.0029	0.0161	0.022
KEGG_WNT_SIGNALING_PATHWAY	0.3771	2.2663	0.0028	0.0160	0.022
KEGG_SYSTEMIC_LUPUS_ERYTHEMATOSUS	-0.3203	-2.0683	0.0027	0.0160	0.022
KEGG_ECM_RECEPTOR_INTERACTION	0.3551	2.0660	0.0027	0.0160	0.022
KEGG_CELL_ADHESION_MOLECULES_CAMS	0.2806	2.0478	0.0032	0.0164	0.022
KEGG_MELANOMA	0.4289	1.9709	0.0026	0.0160	0.022
KEGG_GAP_JUNCTION	0.3660	1.9673	0.0027	0.0160	0.022
KEGG_COMPLEMENT_AND_COAGULATION_CASCADES	0.3140	1.9254	0.0085	0.0321	0.044
KEGG_FOCAL_ADHESION	0.2546	1.8580	0.0032	0.0164	0.022
KEGG_REGULATION_OF_ACTIN_CYTOSKELETON	0.2420	1.7558	0.0095	0.0349	0.048

**Table 2:** The information of reactome pathways from top 20 GSEA enrichment analysis.

Term	ES	NES	NOM p-val	FDR q-val	FWER p-val
REACTOME_FORMATION_OF_THE_CORNIFIED_ENVELOPE	-0.5825	-3.7616	0.0014	0.0132	0.018
REACTOME_KERATINIZATION	-0.4507	-3.2360	0.0013	0.0132	0.018
REACTOME_HDACS_DEACETYLATE_HISTONES	-0.5251	-2.8844	0.0015	0.0132	0.018
REACTOME_HATS_ACETYLATE_HISTONES	-0.5094	-2.8126	0.0015	0.0132	0.018
REACTOME_HCMV_INFECTION	-0.4414	-2.6344	0.0015	0.0132	0.018
REACTOME_FCERI_MEDIATED_MAPK_ACTIVATION	-0.4736	-2.6146	0.0015	0.0132	0.018
REACTOME_RHO_GTPASES_ACTIVATE_PKNS	-0.4734	-2.6002	0.0015	0.0132	0.018
REACTOME_HCMV_EARLY_EVENTS	-0.4440	-2.5984	0.0015	0.0132	0.018
REACTOME_GENE_SILENCING_BY_RNA	-0.4684	-2.5728	0.0015	0.0132	0.018
REACTOME_CHROMATIN_MODIFYING_ENZYMES	-0.4369	-2.5048	0.0015	0.0132	0.018
REACTOME_FCGR_ACTIVATION	-0.4469	-2.4671	0.0015	0.0132	0.018
REACTOME_M_PHASE	-0.4115	-2.3866	0.0015	0.0132	0.018
REACTOME_ESTROGEN_DEPENDENT_GENE_EXPRESSION	-0.4285	-2.3534	0.0015	0.0132	0.018
REACTOME_CREATION_OF_C4_AND_C2_ACTIVATORS	-0.4144	-2.2880	0.0015	0.0132	0.018
REACTOME_FCGR3A_MEDIATED_IL10_SYNTHESIS	-0.3940	-2.2592	0.0015	0.0132	0.018
REACTOME_FCERI_MEDIATED_CA_2_MOBILIZATION	-0.4014	-2.2583	0.0015	0.0132	0.018
REACTOME_SIGNALING_BY_THE_B_CELL_RECEPTOR_BCR	-0.3831	-2.2421	0.0015	0.0132	0.018
REACTOME_AMYLOID_FIBER_FORMATION	-0.3830	-2.2089	0.0015	0.0132	0.018
REACTOME_FC_EPSILON_RECEPTOR_FCERI_SIGNALING	-0.3573	-2.1204	0.0015	0.0132	0.018
REACTOME_CELLULAR_RESPONSES_TO_EXTERNAL_STIMULI	-0.2940	-2.0762	0.0013	0.0132	0.018

**Table 3:** The information of wiki pathways from top 20 GSEA enrichment analysis.

Term	ES	NES	NOM p-val	FDR q-val	FWER p-val
WP_CILIOPATHIES	0.4882	2.9928	0.0028	0.0160	0.022
WP_ECTODERM_DIFFERENTIATION	0.4290	2.8038	0.0029	0.0161	0.022
WP_HISTONE_MODIFICATIONS	-0.5835	-2.4118	0.0016	0.0132	0.018
WP_HIPPOMERLIN_SIGNALING_DYSREGULATION	0.3701	2.3867	0.0028	0.0160	0.022
WP_WNT_SIGNALING	0.4045	2.3533	0.0027	0.0160	0.022
WP_MIRNA_TARGETS_IN_ECM_AND_MEMBRANE_RECEPTORS	0.5139	2.3469	0.0025	0.0160	0.022
WP_PATHWAYS_REGULATING_HIPPO_SIGNALING	0.4142	2.1786	0.0027	0.0160	0.022
WP_CARDIAC_PROGENITOR_DIFFERENTIATION	0.4024	2.1629	0.0027	0.0160	0.022
WP_MESODERMAL_COMMITMENT_PATHWAY	0.3456	2.1464	0.0058	0.0246	0.033
WP_PRADERWILLI_AND_ANGELMAN_SYNDROME	0.4248	2.1443	0.0026	0.0160	0.022
WP_LNCRNA_INVOLVEMENT_IN_CANONICAL_WNT_SIGNALING_AND_COLORECTAL_CANCER	0.4034	2.1196	0.0026	0.0160	0.022
WP_ENDODERM_DIFFERENTIATION	0.3532	2.0697	0.0028	0.0160	0.022
WP_ESC_PLURIPOTENCY_PATHWAYS	0.3566	1.9728	0.0027	0.0160	0.022
WP_INTEGRATED_BREAST_CANCER_PATHWAY	0.4261	1.9580	0.0026	0.0160	0.022
WP_CILIARY_LANDSCAPE	0.4854	1.9211	0.0075	0.0306	0.042
WP_COMPLEMENT_AND_COAGULATION_CASCADES	0.3396	1.9004	0.0027	0.0160	0.022
WP_SUDDEN_INFANT_DEATH_SYNDROME_SIDS_SUSCEPTIBILITY_PATHWAYS	0.2521	1.8529	0.0032	0.0164	0.022
WP_PRIMARY_FOCAL_SEGMENTAL_GLOMERULOSCLEROSIS_FSGS	0.3458	1.7949	0.0076	0.0308	0.042
WP_PI3KAKT_SIGNALING_PATHWAY	0.2071	1.7724	0.0081	0.0316	0.043
WP_FOCAL_ADHESIONPI3KAKTMTORSIGNALING_PATHWAY	0.2055	1.6856	0.0079	0.0314	0.043

## Discussion

Cancer diagnosis, treatment, and prevention have always been hot topics in medical research, and in recent years pan-cancer analysis has gained much attention, which is more reflective of the cancer panorama. The unexpected discovery of new effective prognostic markers or therapeutic targets can help to reduce cancer mortality and improve survival rates. For example, in mucoepidermoid carcinoma, the discovery of PCP4/PEP19 and HER2 as novel prognostic markers is conducive to solving the problem of poor prognosis of cancer and can also be used in molecular-targeted therapies (31). At the same time, some molecular subtypes of tumors cannot be explained by standard clinical parameters or commonly used biomarkers; the emergence of the renin–angiotensin system genes as a new prognostic marker of acute myeloid leukemia (AML) may solve this problem (32). The field of tumor markers deserves to be researched continuously. Therefore, in the study, we aimed to explore the feasibility of *EPOR* as a prognostic marker and its role in tumor immunity, tumor occurrence, and progression through bioinformatics techniques.

EPOR is present not only in hematopoietic cells but also in non-hematopoietic cells such as neurons (10), endothelial cells (11), and skeletal muscle cells (33) and in various tumors such as breast cancer (34) and head and neck cancer (35). Regarding EPOR expression in cancers, on the one hand, it is believed that EPOR expression is upregulated, such as in prostate cancer (21) and glioma (22); on the other hand, no significant EPOR expression was detected in tumor cell lines and solid tumor specimens, and Swift et al. (36) suggested that low levels of EPOR expression are common in tumor cell lines, Elliott et al. (37) questioned the assumption that most tumors express high levels of functional EPOR proteins, and Patterson et al. (38) did not detect the utilization of the functional EPOR pathway in primary tumor cells isolated from tumor tissues such as human breast cancer. In the study, TCGA database showed that *EPOR* expression was upregulated in CHOL, LIHC, STAD, and THCA, which was consistent with the results of previous studies (19, 20, 23, 24); in addition, we found that *EPOR* expression was upregulated in BLCA, HNSC, and KIRC and downregulated in LUAD and LUSC. After combining the GTEx database, *EPOR* was found to be expressed at lower levels in 18 cancer tissues than normal tissues, including BRCA, CESC, and COAD, and at higher levels in CHOL, GBM, HNSC, LGG, SKCM, and TGCT. The CCLE database showed that *EPOR* expression was highest in the LAML cell lines and lowest in the HNSC cell lines, and in the specific cell line, *EPOR* expression was highest in the MOLM.16 cell line of LAML and lowest in the HCC1187 cell line of BRCA. Furthermore, we found significant differences in *EPOR* expression in different tumor stages of BLCA, KICH, and PAAD, with enhanced specificity in erythroid cells, hepatic stellate cells, and Hofbauer cells. Its low tissue specificity and low cell line specificity were further evidence of its widespread distribution.

EPO plays a role in promoting proliferation in hematopoietic progenitor cells and may also promote the growth of tumor cells, thus promoting tumor progression and metastasis, leading to poor prognosis of patients. Moreover, the effect of EPOR, as the receptor of EPO, on the prognosis of cancer patients has been discussed. On the one hand, a high EPOR expression in locally advanced squamous cell carcinoma of the head and neck was considered to be an independent prognostic factor for OS and was associated with poorer OS (25); this was similarly concluded in patients with oral squamous carcinoma, where a high EPOR expression was associated with aggressive tumor behavior and poorer prognosis (26). The activation of EPOR in melanoma was thought to promote tumor progression and contributed to survival of tumor cells (39); inhibition of *EPOR* gene expression in non-small cell lung cancer (NSCLC) reduced the growth of NSCLC cells under hypoxia (40). On the one hand, there was no significant difference in survival rates between patients with different EPOR expression in gastric and cervical carcinoma (19, 27). On the other hand, high levels of *EPOR* mRNA in myeloma were associated with a better prognosis (30); recurrence-free survival was significantly improved in ER +/EPOR + breast cancer patients with untreated tamoxifen in breast cancer (41), and in the breast cancer cell lines, RAMA 37 cells (low *EPOR* expression) had a stronger proliferation ability than RAMA 37-28 cells (high *EPOR* expression), suggesting that high *EPOR* expression can reduce the ability of cells to divide (42).

In our results, OS analysis showed that a high *EPOR* expression was associated with a high survival rate in LUAD, which was consistent with Rózsás et al. (29), and a high *EPOR* expression was associated with low survival in KIRC, which may contradict the findings of Szendrői et al. (28). We also found that a high *EPOR* expression was negatively correlated with the prognosis of COAD and MESO and positively correlated with the prognosis of PAAD. PFS analysis showed that a high *EPOR* expression was negatively correlated with the prognosis of CHOL, COAD, LUSC, MESO, PRAD, and TGCT and positively correlated with the prognosis of BLCA and PAAD. DSS analysis showed that a high *EPOR* expression was negatively correlated with the prognosis of MESO and positively correlated with the prognosis of BLCA and BRCA. DFS analysis showed that a high *EPOR* expression was negatively correlated with the prognosis of CHOL, LUSC, PRAD, and TGCT and positively correlated with the prognosis of ACC and PAAD. PFI analysis showed that a high *EPOR* expression was negatively correlated with the prognosis of CESC, LUSC, and MESO and positively correlated with the prognosis of BLCA and LUAD. Meanwhile, we also found that *EPOR* had better predictive ability for the prognosis of COAD, LUAD, MESO, and PAAD. These findings suggest that *EPOR* has the potential as an effective biomarker.

We also found that EPOR-interacting proteins include JAK2, EPO, and STAT5A/B, MAPK1/3, which fully suggests that EPOR interacts with EPO and is closely related to the activation of JAK2, STAT5, and MAPK (3, 4, 6). *EPOR* is associated with numerous diseases such as urological disorders, musculoskeletal or connective tissue disorders, immune system disorders, endocrine system disorders, hematologic disorders, cancer, or benign tumors, reflecting its broad distribution, and it plays a key role in the human body. As small non-coding RNAs of approximately 21–23 nt, miRNAs regulate gene expression posttranscriptionally through suppressing mRNA translation or inducing mRNA degradation by hybridizing to the 3′-untranslated regions (3′-UTR) of mRNAs (43). We found that *EPOR* may be the target genes of hsa-miR-575, hsa-miR-5011-5p, hsa-miR-503-5p, etc.

The tumor microenvironment is the survival environment for tumor cells to proliferate and metastasize in deep tissues, containing tumor cells, immune cells, stromal cells, and a variety of active molecules, which plays a key role in tumor progression (44). Numerous studies have shown that *EPOR* is expressed on a variety of immune cells including macrophages, dendritic cells, mast cells, and lymphocytes (45, 46), and it can connect the innate and adaptive immune systems, mediating the strong direct immunomodulatory effect of EPO on immune cells (47). In the study, *EPOR* expression correlated with some extent with immune cell infiltration. For the six most common immune cells, *EPOR* expression correlated more strongly with CD4 + T cell infiltration in ACC (r = 0.357), KIRP (r = 0.418), and READ (r = 0.34). *EPOR* expression correlated more strongly with macrophage infiltration in MESO (r = 0.356) and READ (r = 0.307). *EPOR* expression correlated more strongly with neutrophil infiltration in COAD (r = 0.355). Moreover, *EPOR* expression correlated more strongly with DC infiltration in COAD (r = 0.329), ESCA (r = 0.323), LIHC (r = 0.309), and UVM (r = 0.32). In addition, *EPOR* expression in PCPG correlated more strongly with tumor purity, and the correlation was stronger (r = 0.351). ImmuneScore, StromalScore, and ESTIMATEScore were used to evaluate TME; in this study, *EPOR* expression in ACC (r = - 0.446), CESC (r = - 0.323), PCPG (r = - 0.315), and TGCT (r = - 0.336) correlated more strongly with ImmuneScore; expression in ACC (r = - 0.411), PCPG (r = - 0.333), and TGCT (r = - 0.307) correlated more strongly with ESTIMATEScore, and expression in ESCA (r = 0.303), MESO (r = 0.371), READ (r = 0.301), and PCPG (r = - 0.320) showed stronger correlations with StromalScore.

Among the more than 30 immune checkpoints we studied, more than 2/3 of the immune checkpoint genes were associated with *EPOR* expression in ESCA, LIHC, READ, THCA, and UVM. For the five cancers associated with OS, *EPOR* expression in COAD was positively associated with 27 immune checkpoint genes, and that in KIRC was associated with 19 immune checkpoint genes. Among the 33 tumors, *TNFRSF14* was significantly associated with *EPOR* expression in 20 cases and *ADORA2A* was significantly associated with *EPOR* expression in 22 cases. Among the 41 chemokines we studied, *EPOR* expression correlated with CXCL2–3, CXCL13, and CCL17/19/22 in CESC, CCL15 in BLCA, CXCL14 in COAD, CXCL2/5 in ESCA, CXCL16 in KIRC, CCL15 in KIRP, CX3CL1 and CXCL12 in PCPG, and CCL24 in TGCT, and the correlation was strong (|r| ≥ 0.3, p < 0.001). It has been claimed that myeloid-derived suppressor cells (MDSCs), which are precursors of DCs, macrophages, or granulocytes, have the ability to negatively regulate the immune response in cancer, with a subpopulation of monocytic MDSCs mediated by the EPOR-mediated Jak2/GATA3/STAT3 pathway induced (48). All this evidence suggests that EPOR, like EPO, may play a non-negligible role in tumor immunity.

Mutations in *EPOR* are very rare (49), and the same conclusion was obtained in the study. The average frequency of *EPOR* alterations in pan-cancer was only 1.9%, but we unexpectedly found that *EPOR* gene alterations in pan-cancer prolonged DFS in patients (p = 8.53e-4) and that the frequency of *EPOR* alterations in OV was higher, at around 8%. Numerous studies have shown that TMB can be used as a prognostic marker for tumor immunotherapy, such as BLCA, colorectal cancer, NSCLC, and HNSC (50–52). MSI is classified as high instability (MSI-H), low instability (MSI-L), and stable (MS-S), and MSI has been also reported as a prognostic marker (53). The MMR genes mainly repair base mismatches during DNA replication, and mutations cause a decrease in DNA stability, resulting in microsatellite instability and consequent accumulation of mutations, leading to malignancy. MMR status has been shown to be moderately consistent with MSI status and to be also an independent prognostic factor (54). DNA methylation is actually the beginning stage of cancer, and DNA methylation profiling is an emerging tool that will serve as an aid to improve the accuracy of cancer diagnosis (55). In the study, *EPOR* expression was positively correlated with TMB of COAD, SARC, and SKCM and negatively correlated with TMB of BRCA, CESC, KIRP, LIHC, and PAAD. Moreover, *EPOR* expression was positively correlated with MSI of ACC, COAD, LGG, LUAD, LUSC, PAAD, and PRAD and negatively correlated with MSI of KIRC and SARC. *EPOR* expression in BRCA, CESC, HNSC, LIHC, OV, and THYM was significantly correlated with all five MMR genes, *MLH1*, *MSH2*, *MSH6*, *PMS2*, and *EPCAM*, and with four of them in KIRP, PCPG, STAD, TGCT, and UVM. In addition, promoter methylation levels of *EPOR* were higher in COAD, ESCA, KIRC, KIRP, LIHC, LUAD, LUSC, and PAAD cancer tissues and lower in BLCA, BRCA, UCEC, and THCA cancer tissues compared to normal tissues. *EPOR* was significantly associated with three DNA methyltransferases, DNMT1, DNMT3A, and DNMT3B, and two RNA methyltransferases, METTL3 and CMTR1, in more than 2/3 of tumors. In the study, our enrichment analysis of the interacting proteins of EPOR and co-expressed genes of *EPOR* revealed that EPOR can be involved in biological processes such as peptide hormone response, regulation of MAPK activity, ERK1 and ERK2 cascade, JAK-STAT cascade, and regulation of PI3K signaling. In addition to JAK-STAT, PI3K-Akt, and MAPK signaling pathways, it is also closely related to Ras, chemokines, and neurotrophic factor signaling pathways, as well as natural killer cell cytotoxic activity and cancer pathways. In addition, DEGs of *EPOR* were significantly enriched in pathways such as axon guidance, Wnt signaling pathway, ECM receptor interactions, cell adhesion molecules, and abnormal regulation of Hippo Merlin signaling.

To our knowledge, this study is the first pan-cancer analysis of *EPOR*, demonstrating that *EPOR* plays an important role in the occurrence and progression of tumors and is expected to be an important prognostic marker for specific cancers. This study resolves the controversy of whether *EPOR* is highly or lowly expressed in cancer. In addition, the results of this study indicate that *EPOR* plays a non-negligible role in tumor immunity, which provides a new direction for tumor research. However, there are still some limitations in this study. Our study is based on bioinformatics analysis, and the results will be more convincing if combined with experimental validation such as immunohistochemistry or prospective studies of large clinical samples. In addition, in our findings, *EPOR* was a protective factor in a subset of specific cancers and a risk factor in another subset of cancers, but the mechanism of action of *EPOR* in different cancers needs to be further explored.

## Data Availability Statement

The original contributions presented in the study are included in the article/**Supplementary Material**. Further inquiries can be directed to the corresponding author.

## Author Contributions

YZ proposed the research idea and designed the study with SW. YZ and SW collected the data. SH performed the analysis. YZ drafted the manuscript. YF critically revised the manuscript. All authors contributed to the article and approved the submitted version.

## Conflict of Interest

The authors declare that the research was conducted in the absence of any commercial or financial relationships that could be construed as a potential conflict of interest.

## Publisher’s Note

All claims expressed in this article are solely those of the authors and do not necessarily represent those of their affiliated organizations, or those of the publisher, the editors and the reviewers. Any product that may be evaluated in this article, or claim that may be made by its manufacturer, is not guaranteed or endorsed by the publisher.
