# Supplementary figures and images for: Pan-Cancer Analysis Based on EPOR Expression With Potential Value in Prognosis and Tumor Immunity in 33 Tumors
Source: Front Oncol. 2022 Mar 14;12:844794. doi: 10.3389/fonc.2022.844794 (PMC8963997; doi:10.3389/fonc.2022.844794)

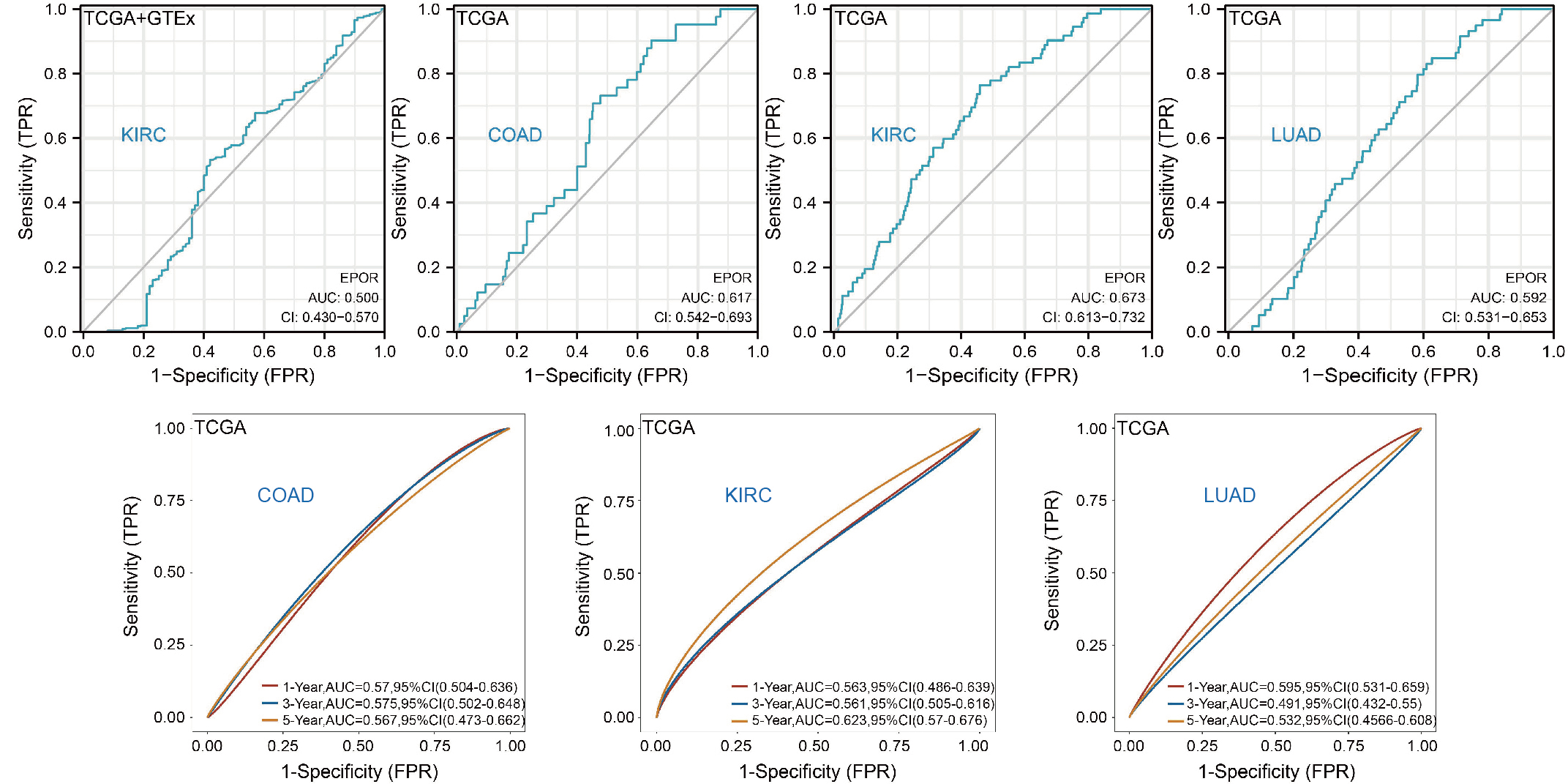

Supplement: Supplementary file 1 [file DataSheet_1.zip › Supplementary figure 1.jpg]

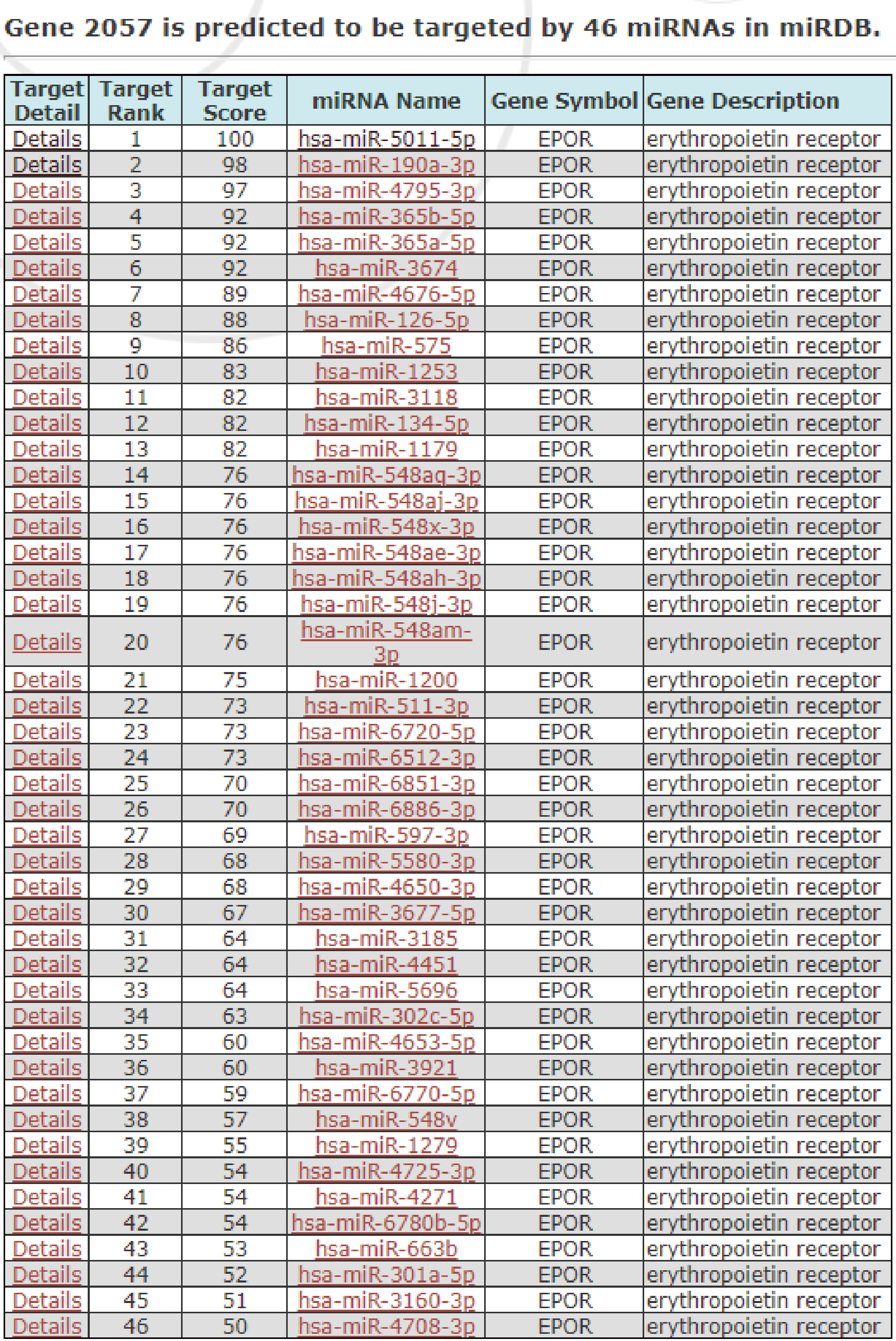

Supplement: Supplementary file 1 [file DataSheet_1.zip › Supplementary Figure 2.jpg]

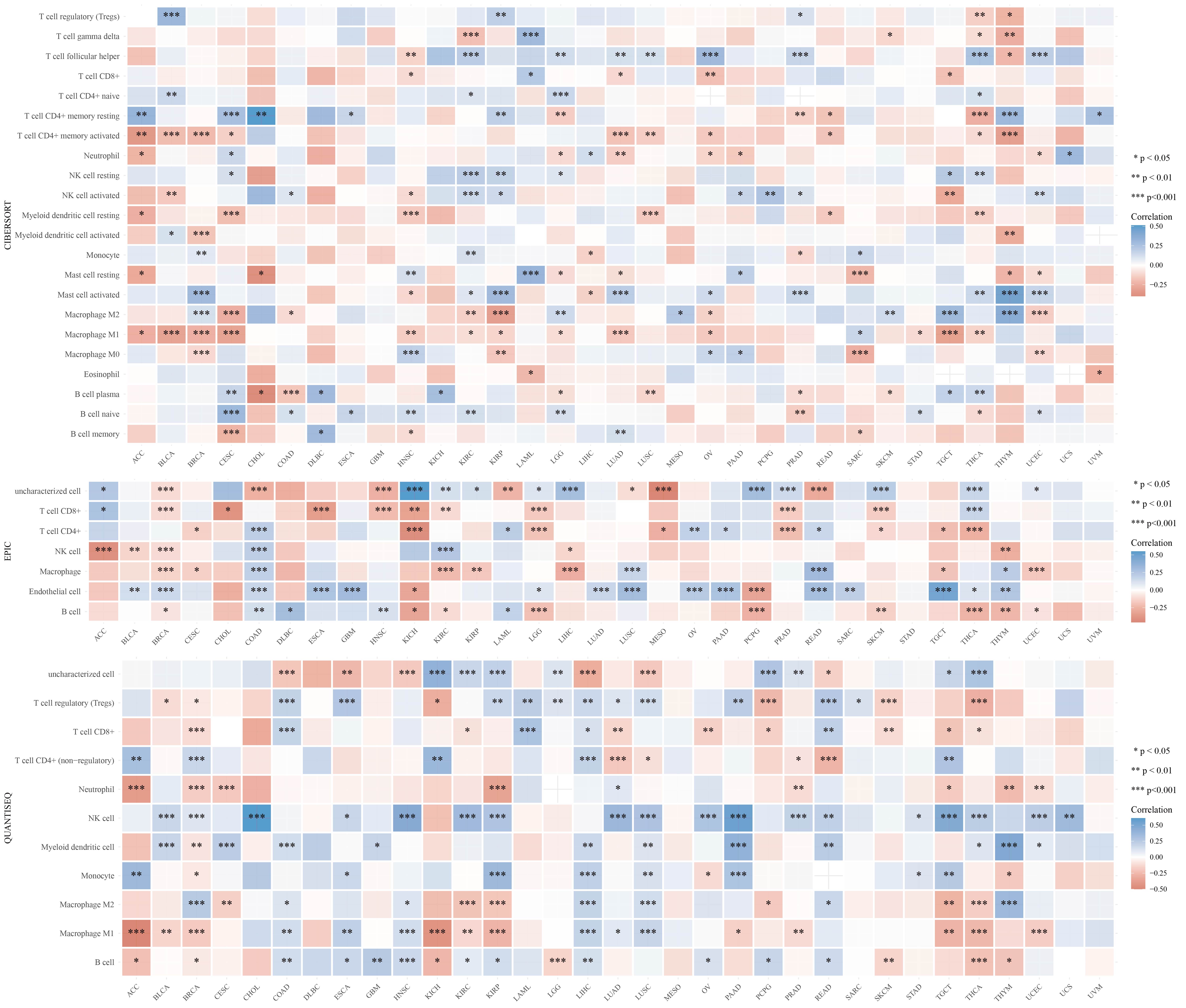

Supplement: Supplementary file 1 [file DataSheet_1.zip › Supplementary Figure 3.jpg]

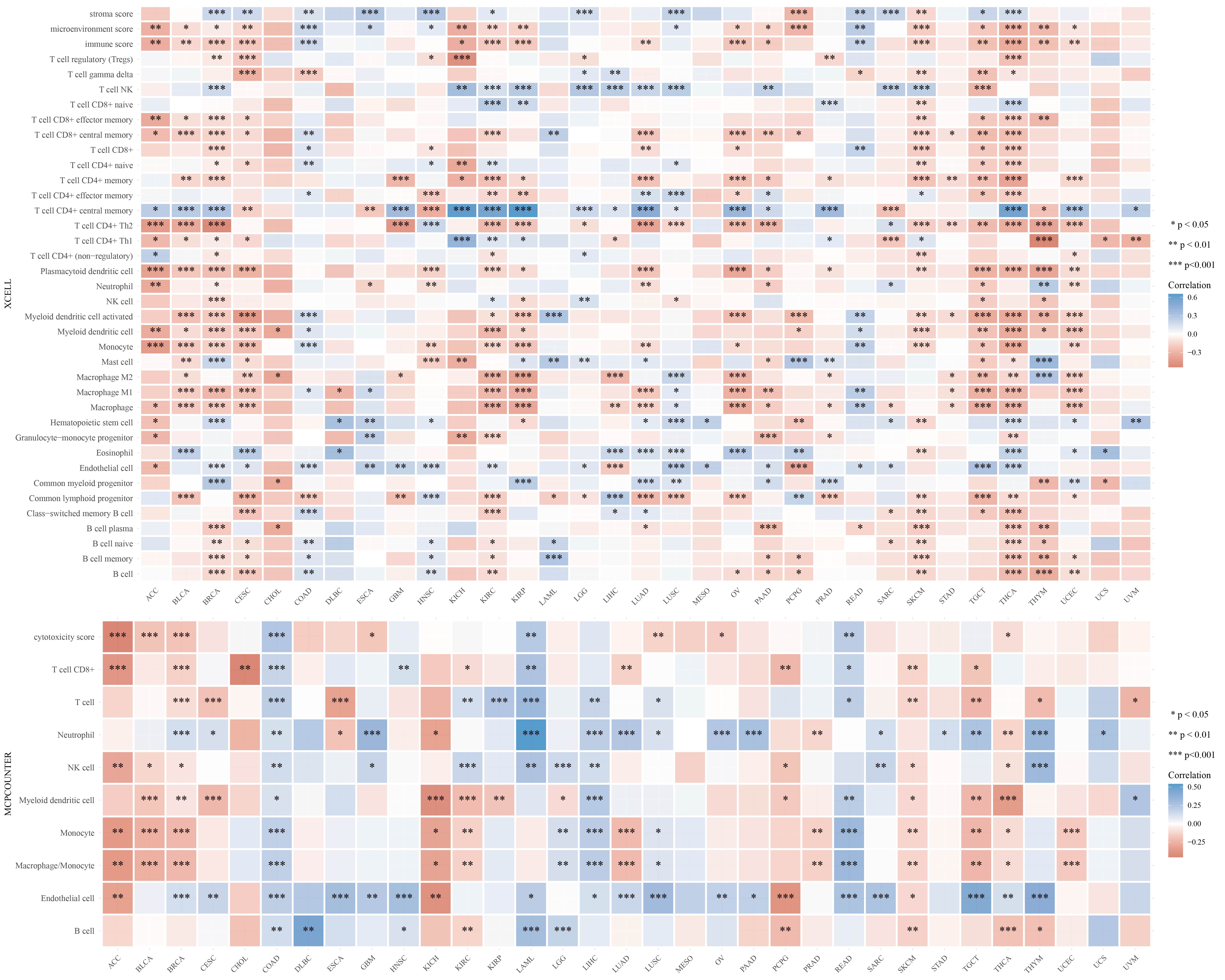

Supplement: Supplementary file 1 [file DataSheet_1.zip › Supplementary Figure 4.jpg]
